# Whole Genome Sequencing of Enteroviruses Species A to D by High-Throughput Sequencing: Application for Viral Mixtures

**DOI:** 10.3389/fmicb.2018.02339

**Published:** 2018-09-28

**Authors:** Marie-Line Joffret, Patsy M. Polston, Richter Razafindratsimandresy, Maël Bessaud, Jean-Michel Heraud, Francis Delpeyroux

**Affiliations:** ^1^Unité de Biologie des Virus Entériques, Institut Pasteur, Paris, France; ^2^Institut National de la Santé et de la Recherche Médicale, Paris, France; ^3^WHO Collaborating Center for Research on Enteroviruses and Viral Vaccines, Institut Pasteur, Paris, France; ^4^Unité de Virologie, Institut Pasteur de Madagascar, Antananarivo, Madagascar

**Keywords:** human enteroviruses, whole-genome sequences, high-throughput sequencing, viral mixtures, enterovirus identification

## Abstract

Human enteroviruses (EV) consist of more than 100 serotypes classified within four species for enteroviruses (EV-A to -D) and three species for rhinoviruses, which have been implicated in a variety of human illnesses. Being able to simultaneously amplify the whole genome and identify enteroviruses in samples is important for studying the viral diversity in different geographical regions and populations. It also provides knowledge about the evolution of these viruses. Therefore, we developed a rapid, sensitive method to detect and genetically classify all human enteroviruses in mixtures. Strains of EV-A (15), EV-B (40), EV-C (20), and EV-D (2) viruses were used in addition to 20 supernatants from RD cells infected with stool extracts or sewage concentrates. Two overlapping fragments were produced using a newly designed degenerated primer targeting the conserved CRE region for enteroviruses A-D and one degenerated primer set designed to specifically target the conserved region for each enterovirus species (EV-A to -D). This method was capable of sequencing the full genome for all viruses except two, for which nearly 90% of the genome was sequenced. This method also demonstrated the ability to discriminate, in both spiked and unspiked mixtures, the different enterovirus types present.

## Introduction

The common human viruses, human enteroviruses (EV), consist of more than 100 serotypes most classified within four species for enteroviruses (EV-A to -D) and three species for rhinoviruses. Enteroviruses are ubiquitous and resilient in the environment and primarily transmitted fecal-orally. Human enteroviruses have been implicated in a variety of human diseases, including the common cold, hand foot and mouth disease, acute hemorrhagic conjunctivitis, myocarditis, encephalitis, and poliomyelitis.

Enteroviruses are non-enveloped viruses, approximately 7500 nucleotides (nt) in length with a positive, single-stranded RNA genome. There are two untranslated regions (5′ and 3′ -UTR) flanking a large open reading frame encoding a polyprotein that is cleaved to give three precursors P1 to P3. These precursors are subsequently cleaved to give functional proteins: (1) P1 giving rise to four structural capsid proteins (VP1–VP4) and (2) P2 and P3, the non-structural proteins involved in the virus life cycle.

Studies have been conducted to best determine the most accurate way to identify and classify enteroviruses, giving rise to some “gold standards” for detection and typing. Typically, enteroviruses have been isolated on a variety of cell lines (e.g., RD, GMK, Vero, CaCo-2, L20B, HEp2c, and HeLa) based on their ability to propagate and show cytopathic effect (CPE), followed by serotyping using neutralization assays (Silva et al., 2008; Poh and Tan, 2010; Blomqvist and Roivainen, 2016). Despite cell amplification being an appropriate method, it is laborious, time-consuming, and expensive (Nijhuis et al., 2002). It has been shown that there is a good correlation between the sequences of the VP1 nucleotidic and amino acid sequences and enterovirus serotypes (Oberste et al., 1999a; Caro et al., 2001). Thus, VP1 sequences have been used as a gold standard for typing and, if relevant, subtyping enteroviruses (Oberste et al., 1999a,b; Nix et al., 2006; Poh and Tan, 2010).

In addition to typing, studies dedicated to evaluating the enterovirus genomic diversity have shown that besides mutations, intra- and inter-typic genetic recombination is also a frequent mechanism of viral evolution (Lukashev et al., 2003; Simmonds, 2006; Combelas et al., 2011; Joffret et al., 2012). Enterovirus genomes frequently display mosaicism due to genetic exchanges among different enterovirus strains and types. Since this kind of genetic diversification can be at the source of genotypic and phenotypic diversity (Sadeuh-Mba et al., 2013; Bessaud et al., 2016) it is essential to determine the whole genomic sequences of enteroviruses for surveillance and public health purposes, as well as for basic research.

For obtaining sequencing data, the traditional Sanger method is capable of sequencing the whole genome but it is time-consuming and it cannot simultaneously sequence a mixture of viruses; thereby, making a large-scale surveillance project difficult to conduct due to the presence of many viruses. Furthermore, it makes it challenging to specifically target every known enterovirus and impossible to identify any unknown viruses.

Next generation sequencing (NGS) methods offer a new powerful sequencing tool for the identification and characterization of enteroviruses. This has been successfully used to sequence partial or whole genome sequences of poliovirus and of enteroviruses species C (Bessaud et al., 2016; Montmayeur et al., 2017; Sahoo et al., 2017). Additionally, a generic assay for whole genome amplification and deep sequencing of enterovirus A71 was published (Tan et al., 2015). Despite advances in molecular methods, none of these assays were designed to simultaneously amplify the whole genome for all four enterovirus species, and it is known that mixtures of enteroviruses can be found in human stool or sewage. Hence, the goal of this research was to design generic and species-specific primers in order to develop an assay capable of viral typing and sequencing the whole genome for all enteroviruses of species A to D present in samples containing viral mixtures.

## Materials and Methods

### Viruses

For this study 15-EVA, 40-EVB, 20-EVC, and 2-EVD viruses were used (**Table 1**) to test and analyze whole-genome sequencing. The prototype strains selected were from the European Virus Archive (EVAg)^1^. Additionally, we used 20 enterovirus positive supernatants from human rhabdomyosarcoma cells (RD cells) from the Madagascar National Polio Laboratory. These cells were infected with either 10 human stool extracts or 10 sewage concentrates collected during poliomyelitis surveillance in Madagascar and prepared according to WHO protocols (World Health Organization Polio Laboratory manual, 4th edition, 2004). Infections of RD cells with these extracts were followed by full cytopathogenic effect, resembling that induced by enteroviruses.

**Table 1:** Viruses used in this study.

Type	Strain or isolate	*n*° Databank	Consensus length	Type	Strain or isolate	*n*° Databank	Consensus length
**SPECIES A**				**SPECIES B continued**			
CV-A2	Fleetwood	AY421760	7411	E-11	RO-91-91	AJ577594	7460
CV-A3	Olson	AY421761	7411	E-12	RO-78-3-74	LS451298	7351
CV-A6	Gdula	AY421764	7551	E-13	DelCarmen	AY302539	7410
CV-A6	MAD-2628-11	LT719047	7503	E-14	Tow	AY302540	7352
CV-A7	Parker	AY421765	7395	E-14	RO-81-1-79	LS451299	7457
CV-A10	Kowalik	AY421767	7420	E-15	CH-96-51	AY302541	7364
CV-A10	MAD-9856-11	LT719059	7402	E-20	JV-1	AY302546	7321
CV-A10	MAD-3995-11	LT719056	7415	E-21	Farina	AY302547	7000
CV-A12	Texas-12	AY421768	7623	E-24	DeCamp	AY302548	7239
CV-A14	G14	AY421769	7540	E-25	JV-4	AY302549	7443
CV-A14	MAD-2718-11	LT719062	7521	E-26	Coronel	AY302550	7459
CV-A16	G10	U05876	7000	E-27	Bacon	AY302551	7339
EV-A71	MAD-3126-11	LT719063	7400	E-29	JV-10	AY302552	7362
EV-A71	MAD-72341-04	LT719065	7326	E-30	Bastianni	AY311938	7440
EV-A71	CAE-146-08	LT719066	7511	E-32	PR-10	AY302555	7448
**SPECIES B**				EV-B69	Toluca-1	AY302560	7338
CV-A9	RO-609-4-80	LS451285	7398	**SPECIES C**			
CV-B1	RO-98-1-74	LS451286	7410	CV-A1	Tompkins	AF499635	7351
CV-B2	Ohio	AF081485	7350	CV-A11	Belgium	AF499636	7326
CV-B3	RO-123-1-95	LS451287	7519	CV-A11	MAD-66122	JF260917	7296
CV-B3	RO-69-1-89	LS451288	7327	CV-A11	MAD-66990	JF260918	7000
CV-B4	E2	AF311939	7173	CV-A13	Flores	AF465511	7368
CV-B4	RO-69-1-86	LS451289	7351	CV-A13	G13	AF499640	7380
CV-B5	RO-14-5-70	LS451290	7273	CV-A13	MAD-67001	JF260920	7298
CV-B5	Faulkner	AF114383	7404	CV-A13	MAD-67900	JF260921	7000
CV-B6	Schmitt	AF039205	7209	CV-A17	G12	AF499639	7301
CV-B6	RO-86-1-73	LS451291	7345	CV-A17	MAD-67610	JF260924	7368
E-1	Farouk	AF029859	7313	CV-A17	MAD-68154	JF260925	7541
E-1	RO-122-1-74	LS451292	7351	CV-A19	8663	AF499641	7449
E-3	Morrissey	AY302553	7366	CV-A20	IH35	AF499642	7415
E-4	Pesacek	AY302557	7359	CV-A20a	Tulane	X87601	7305
E-5	Noyce	AF083069	7340	CV-A20b	Cecil	X87602	7360
E-5	RO-79-2-71	LS451293	7444	CV-A21	Coe	D00538	7393
E-6	D’Amori	AY302558	7436	CV-A24	DOU-054	JX417873	7366
E-6	RO-24-9-79	LS451294	7421	EV-C95	T08-083	JX417822	6895
E-7	RO-434-2-81	LS451295	7355	EV-C99	MAD-69412-03	LS451300	7438
E-7	RO-141-2-95	LS451296	7386	EV-C99	MAD-69558-03	LS451301	7383
E-9	Hill	X84981	7379	**SPECIES D**			
E-9	RO-116-6-82	LS451297	7369	EV-D68	Fermon	AY426531	7369
E-11	Gregory	X80059	7564	EV-D70	J670/71	D00820	6467

For the collection of stool from Madagascar, the protocol was approved by the National Ethics Committee of the Ministry of Public Health in Madagascar (Agreement N° 22-MSANP/CE). Parental written informed consent was given.

### Primer Design

**FIGURE 1:**
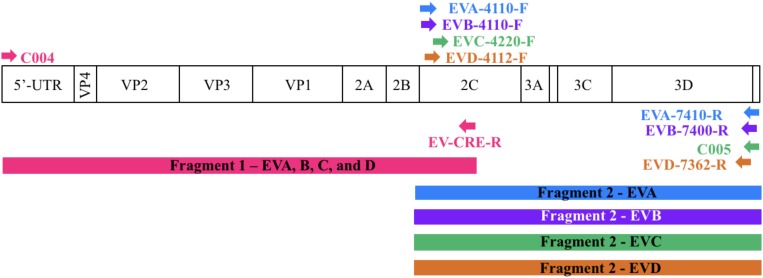
Location of primers used in this study. The diagram shows the organization of the enterovirus genome. Arrows indicate the sites targeted by the proposed primers. The RT-PCR products are colored coded to represent the corresponding primer pair. The first half of the genome (fragment 1) is amplified using one primer pair whereas the second half (fragment 2) is amplified using primers targeting each enterovirus species.

For each enterovirus group, complete genome sequences were separately aligned using CLC Main Workbench. To achieve the two overlapping fragments, the first half of the genome was amplified using the previously described C004 primer (Bessaud et al., 2016) in combination with a newly designed degenerated primer targeting the conserved CRE (*cis-acting* replication element) region for enteroviruses A-D. The second half of the genome was amplified using one degenerated primer set designed to specifically target the conserved region for each enterovirus species. Therefore, we performed five PCR mixes for each isolate and we pooled the PCR products for sequencing. All primers used in this study are described in **Table 2** and **Figure 1** illustrates the primer locations.

**Table 2:** Primers used for this study.

Primer set	Enterovirus species	Primer name	5′–3′ Sequence	Genome position^1^
1	A, B, C, and D	C004^2^	TTAAAACAGCYYKDGGGTTG	1–20
	A, B, C, and D	EV-CRE-R	CGGBRTTTGSWCTTGAACTG	∼4500
2	A	EVA-4110-F	AARAARTTYAAYGAYATGGC	4110–4130
	A	EVA-7410-R	TTTGCTATTCTGGTTATAAC	7410–7390
	B	EVB-4110-F	GGCGNTGGCTYAARCRAARG	4110–4130
	B	EVB-7400-R	GCACCGAATGCGGAGAATTTAC	7400–7378
	C	EVC-4220-F	GARGCNTGYAAYGCNGCNAARG	4220–4242
	C	C005^2^	CCGAATYAAARRAAAATTTACCC	7437–7415
	D	EVD-4112-F	GGCTAKCMCAAAAGATWGAC	4112–4132
	D	EVD-7362-R	CCAAKTRACCAAAATTTACC	7362–7342

^1^*The specific genome position. Position may vary depending on the strain of the virus. ^*2*^Primer described in Bessaud et al. (2016)*.

### RNA Extraction

Viral RNA was extracted from supernatants of infected cells, using the High Pure Viral RNA kit (Roche Diagnostics, Meylan, France) per manufacturer’s protocol. All RNA was either immediately used for PCR amplification or stored at -80°C for further analysis.

### One-Step RT-PCR Amplification

For entire length genome amplification, we used two primer sets (**Table 2**) per enterovirus group (A, B, C, and D). This allowed us to synthesize the two overlapping amplicons by using the One-Step RT-PCR kit (ref G174 Applied Biological Materials Inc.). The reaction mixture contained 1.5 μl of purified RNA, 12.5 μl of 2× One-step RT-PCR buffer, 1 μl of each forward and reverse primer (20 μM), 0.5 μl of EasyScript RTase (200 U/μl), 1 μl of Bestaq DNA Polymerase (5 U/μl), and 9 μl of DNAse free water. PCR amplification was performed using a thermocycler with the following protocol: 42°C for 30 min, 94°C for 3 min, 35 cycles at 94°C for 30 s, 55°C for 30 s, 72°C for 4 min 30 s, and a last step at 72°C for 10 min, ending with 2 min at 4°C. All PCR products were visualized on ethidium bromide-stained agarose gels to ensure appropriate size products.

The sensitivity of this assay was evaluated using serial fourfold dilutions of four viruses (EV-A71 strain MAD-72341, CV-B4 strain E2, CV-A13 strain MAD 67001, and EV-D 68 strain Fermon) representing each species (**Table 3**). To maintain similar amounts of cellular nucleic acids throughout dilutions, viral stocks were diluted using a supernatant of confluent non-infected HEp-2c cell monolayers frozen and thawed twice and clarified by centrifugation.

**Table 3:** Sensitivity of the NGS assay.

Species (reference strain)^1^	Dilution factor	Viral titer	rRT-PCR cycle threshold^2^	DNA concentration (ng/μL)^3^	Number of reads^4^	Number of reads mapping against the reference (%)^5^	Contig length^6^
EV-A (EV-A71 MAD-72341-04)	4^0^	6.3	18.4	50.6	437 922	434360	7 336
		10e6				(99.2%)	
	4^1^	1.6	21.5	31.8	483 490	471138	7 410
		10e6				(97.5%)	
	4^2^	3.9	23.1	20.1	640 522	565731	7 470
		10e5				(88.3%)	
	4^3^	9.8	30.2	11.7	478 940	357168	7 405
		10e4				(74.6%)	
	4^4^	2.5	31.1	10.2	270 790	109731	7 405
		10e4				(40.5%)	
	4^5^	6.2	29.6	9.5	500 760	85344	7 405
		10e3				(17.0%)	
	4^6^	1.5	32.6	10.6	349 396	24072	7 408
		10e3				(6.9%)	
EV-B (CV-B4 E2)	4^0^	4.0	21.3	58.1	526 474	457529	7 442
		10e7				(86.9%)	
	4^1^	1.0	25.3	36.5	529 664	475725	7 430
		10e7				(89.8%)	
	4^2^	2.5	28.3	22.7	657 448	539309	7 450
		10e6				(82.0%)	
	4^3^	6.3	29.3	15.7	365 820	222881	7 410
		10e5				(60.9%)	
	4^4^	1.6	33.5	15.1	400411	127698	7 434
		10e5				(31.9%)	
	4^5^	3.9	Undeter^7^	17.6	517449	9656	4 294
		10e4				(2.0%)	
	4^6^	9.8	Undeter	10.4	338 670	2375	6 872
		10e3				(0.7%)	
EV-C (CV-A13 MAD 67001)	4^0^	1.0	18.6	29.3	538 236	50299	7 450
		10e8				(93.5%)	
	4^1^	2.5	22.3	12.3	551 962	462287	7 374
		10e7				(83.8%)	
	4^2^	6.3	23.7	10.4	369 904	255970	7 381
		10e6				(69.2%)	
	4^3^	1.6	26.5	9.4	418 630	60056	7 442
		10e6				(14.4%)	
	4^4^	3.9	28.7	10.8	383 840	15414	3 279
		10e5				(4.0%)	
	4^5^	9.8	31.2	10.8	454272	10492	3 371
		10e4				(2.5%)	
	4^6^	2.4	33.6	8.8	467 808	982	3 262
		10e4				(0.2%)	
EV-D (EV-68 Fermon)	4^0^	1.3	24.3	23.8	468 514	465107	7 367
		10e6				(99.3%)	
	4^1^	3.3	28.2	14.1	585 646	494846	7 359
		10e5				(84.5%)	
	4^2^	8.1	32.0	14.2	496 292	408854	7 282
		10e4				(82.4%)	
	4^3^	2.0	35.6	8.3	338 906	178190	7 277
		10e4				(52.6%)	
	4^4^	5.1	Undeter	11.7	547 836	79936	7 359
		10e3				(14.6%)	
	4^5^	1.3	Undeter	7.3	555 258	67439	6 670
		10e3				(12.2%)	
	4^6^	3.2	Undeter	15.1	580 342	11254	3 398
		10e2				(1.9%)	

^1^*The enterovirus species and strain for viruses used in this assay. ^*2*^Cycle threshold values obtained using a pan-enterovirus real-time RT-PCR assay performed on the extracted RNA. ^*3*^Concentration of DNA amplicons obtained by RT-PCR for each dilution. ^*4*^Total number of reads from NGS sequencing. ^*5*^Number of reads mapped against the known reference (indicated in column one) for each viral dilution with percent identity to total reads. ^*6*^The size of each contig (enteroviruses ∼7500 nb). ^*7*^There was no amplification therefore no threshold was obtained*.

This RT-PCR amplification method was developed for the analysis of certain amount of viruses present following amplification in infected cells. It could probably be applied to the direct analysis of human or environmental samples, including different compartment-specific human fluids, provided that the sensitivity of the method has been adapted to the amount of virus present in these samples.

### Virus Mixture Detection

To confirm the ability to detect viruses in a mixture, different samples containing known viral isolates under the following conditions were prepared: (1) equal amounts of four viral isolates representing enterovirus species A, B, C, and D and (2) four viral isolates belonging to enterovirus species B. The viral isolates used to perform these mixture experiments are listed in **Table 4**.

**Table 4:** NGS analysis in three mixtures containing four viruses.

Mixture^1^	Contig name^2^	Contig length^3^	Annotation (virus)^4^	Percent identity^5^	VP1^6^	VP1 length^7^
1	contig 3	6620	Coxsackievirus A10, strain Kowalik	99.9	full	894
	contig 1	7435	Echovirus 11, isolate ROU-9191	99.9	full	876
	contig 8	4681	Coxsackievirus A13, isolate 67001	99.8	partial	343
	contig 2	7318	Enterovirus 68, strain Fermon	99.9	partial	536
2	contig 1	4444	Coxsackievirus B6, strain Schmitt	99.7	full	846
	contig 2	4416	Echovirus 5, strain Noyce	99.9	full	876
	contig 3	4408	Echovirus 7, strain Wallace	99.9	full	876
	contig 4	4384	Coxsackievirus B3, strain Nancy	99.9	full	852
3	contig 1	7416	Echovirus 5, strain Noyce	99.9	full	876
	contig 2	7404	Echovirus 7, strain Wallace	99.9	full	876
	contig 3	7383	Coxsackievirus B6, strain Schmitt	99.9	full	846
	contig 4	7376	Coxsackievirus B3, strain Nancy	99.9	full	852

^1^*Mixture 1 was performed using four viruses (one strain from each species) with equal quantities of RNA. Mixture 2 and 3 were performed using four EV-B viruses. For mixture 2, PCR was performed using primers designed to amplify the first half of the genome, whereas mixture 3 was the PCR for the full genome. ^*2*^The identification of the contig that is provided in the NGS analysis. ^*3*^The length of the contig in nucleotides (∼7500 nt = enterovirus size). ^*4*^The name given for the virus in GeneBank (using BLAST in NCBI). ^*5*^The percent identity between the contig sequence and the nearest sequence of known virus in GeneBank. ^*6*^If the VP1 was retrieved in full or partial size. ^*7*^The length of the VP1 recovered*.

To confirm the ability to detect a mixture of viruses from “real life” conditions, we used 20 supernatants from RD cells which were infected with 10 stool extracts and 10 sewage concentrates, respectively (**Table 5**). RNA extraction, RT-PCR, and NGS sequencing were performed as described in the related sections.

**Table 5:** Detection of viral sequences in supernatants of RD cells infected with stool and sewage samples, using *de novo* assembly.

Sample^1^	Enterovirus contig(s)^2^	VP1 contig(s)^3^	Contig(s) with VP1(nt)^4^	Read Count^5^	Viral type(s)^6^	Enterovirus species^7^
Stool 1	5	1	6886	450016	E-21	EV-B
Stool 2	1	1	7475	557642	E-4	EV-B
Stool 3	3	2	7230	452063	E-11	EV-B
			2069	842	EV-C99	EV-C
Stool 4	2	2	6948	371680	E-2	EV-B
			6932	72007	CV-A4	EV-A
Stool 5	3	1	7247	437921	E-14	EV-B
Stool 6	2	2	7426	363551	E-5	EV-B
			5170	31239	EV-C99	EV-C
Stool 7	5	2	7411	523337	E-13	EV-B
			6605	5567	E-20	EV-B
Stool 8	4	2	7109	605697	EV-B84	EV-B
			1791	75	CV-A4	EV-A
Stool 9	11	2	7581	413225	E-15	EV-B
			1941	359	CV-A13p	EV-C
Stool 10	4	2	7109	339135	E-14	EV-B
			5274	41917	EV-C99	EV-C
SEW 1	3	2	7337	435585	E-6	EV-B
			5095	1657	E-13	EV-B
SEW 2	2	2	4444	297319	E-19	EV-B
			4404	8236	E-7	EV-B
SEW 3	9	5	7403	102527	E-20	EV-B
			7380	141133	E-12	EV-B
			7371	290910	E-7	EV-B
			4094	11083	E-6	EV-B
			3758	390	EV-A76	EV-A
SEW 4	5	3	4574	63109	E-7	EV-B
			3871	13766	CV-B5	EV-B
			7363	390728	E-33	EV-B
SEW 5	9	3	4476	333848	E-11	EV-B
			3302	107812	E-19	EV-B
			2607	4608	E-12	EV-B
SEW 6	8	3	4400	81721	E-6	EV-B
			4367	359419	CV-B5	EV-B
			207	32	E-6p	EV-B
SEW 7	20	2	5980	409716	E-6	EV-B
			2663	1321	E-12	EV-B
SEW 8	3	2	6949	303796	E-12	EV-B
			6858	17635	E-24	EV-B
SEW 9	2	2	4410	56755	E-11	EV-B
			5174	144696	E-6	EV-B
SEW 10	28	7	3838	4963	E-12	EV-B
			6155	13411	E-6	EV-B
			6176	172800	E-6	EV-B
			4228	182570	E-11	EV-B
			683	100	E-6p	EV-B
			1266	227	E-6p	EV-B
			3380	1060	E-33	EV-B

^1^*Type of isolates: RD cells infected by stool extracts or sewage (SEW) concentrates. ^*2*^Number of contigs identified per sample. ^*3*^Number of contigs that contained the VP1 region (needed to identify the virus). ^*4*^Size of the contig with the VP1 region included. Size varies but we are interested in the ones closest to the size of the whole genome (∼7500 nb). ^*5*^Number of reads used to generate the VP1 contigs. ^*6*^Virus serotype (identity) using the VP1 region. Note: p indicates partial size of the VP1 based on the length. In SEW 6 and -10 different E-6 genotypes were found. ^*7*^Enterovirus species classification based on the virus identified*.

### PCR Purification and Next Generation Sequencing (NGS)

For each sample, the five PCR products were pooled and purified using a vacuum method and then sent to the sequencing platform PIBNET (Pasteur International Bioresources Network, Institute Pasteur Paris). The libraries were created using 1 ng of DNA with the Nextera XT DNA Library Preparation Kit in a SureCycler 8800 thermocycler (Agilent). Following purification on AMPure beads (Beckman), the libraries were controlled using the High Sensitivity D1000 assay (Agilent) on a TapeStation 2200. The products were sequenced using Illumina NextSeq HiSeq. All kits were used following manufacturer’s instructions.

### Data Analysis

Using CLC Genomics Workbench 8.5 (CLCbio), we paired and assembled contigs from the raw reads. Next, *de novo* assembly was performed using CLC Main Workbench (CLCbio) with the following parameters: Mismatch cost = 2; Insertion cost = 2; Deletion cost = 2; Length Fraction = 0.5; Similarity Fraction = 0.95. All contigs longer than 200 nt were submitted to National Center for Biotechnology Information (NCBI) for BLAST analysis.

## Results

### Sequence Analysis and Primer Design

To sequence all EVs, we designed primers targeting conserved genomic regions that allowed the synthesis of overlapping amplicons. To amplify the first half of the genome primer C004 (Bessaud et al., 2016) was used in combination with a newly designed generic primer (EV-CRE-R), targeting the CRE region. This primer set was used to amplify the first half of the genome for the four EV species (EV A-D). To ensure the best amplification of the second half of the genome, we designed primers specifically targeting species A, B, C, and D. The combination of the two primer sets (**Table 2**), allowed for the amplification of the 5′ and 3′ parts of the genomes, which led to the synthesis of two overlapping DNA fragments per virus (approximately 400-nt long see **Figure 1**). This method resulted in the amplification of the whole genome for samples containing a single virus or mixture and the amplicon products were used for NGS.

To validate the proposed primer sets capability of generating whole genome sequencing data, we tested 15-EVA, 40-EVB, 20-EVC, and 2-EVD viruses (**Table 1**). We obtained the full genome sequencing data for all viruses except two (EV-C95 T08-083 and EV-D70), for which we obtained 93.6 and 87.5% of the genomic sequences, respectively. In all cases the sequences of the VP1 capsid protein could be used to confirm the type of virus. These results indicated the effectiveness of the proposed primers in conjunction with NGS sequencing to correctly identify the viruses and to reconstruct the whole genome using *de novo* assembly.

Additionally, to evaluate the sensitivity of this assay, we tested a fourfold serial dilution of one representative of each enterovirus species (**Table 3**). For undiluted viruses, the number of reads that mapped against the reference strains ranged between 86.9 to 99.3%, depending on the species. The full-length genome was reconstructed, using *de novo* assembly, for the majority of viruses and dilutions (ranging from 1.0 10e8 to 1.5 10e3 TCID50/ml). For some strains, long contigs overlapping almost the whole genomic sequences were recovered when high amounts of viruses were used (1.6 10e5 for CV-B4 and 1.6 10e6 for CV-A13).

### Mixture Detection

To assess the capacity of the method to detect and identify different enteroviruses in samples containing mixtures, we performed two types of experiments: (1) mixed samples containing different mixtures of known viral isolates under controlled conditions (spiked) and (2) supernatants of cells infected with stool or sewage extracts in which the mixture of viruses was unknown and the conditions were not controlled (unspiked).

#### Spiked Experiments

To simulate true clinical and/or environmental conditions, four representative viral isolates for each enterovirus species were used to make mixture 1 (similar quantities or viral RNA); and four viral isolates of species B were used for mixtures 2 and 3. For all mixtures, RNA was extracted and DNA amplification was performed using the newly designed primer sets.

For mixture 1, we were successful in generating one contig per virus with 99.9% identity with the corresponding sequences in GeneBank using BLAST algorithm (**Table 4**). We were able to recover data about the four viral isolates and to consider the VP1 region used to identify the virus(es) present, either in its full or partial length.

For mixtures 2 and 3 we were able to correctly isolate and identify all four serotypes from species B with 99.7–99.9% identity and we recovered the full length of the VP1 region for the four B viral isolate strains under both conditions (half and full genome amplification). Additionally, for all three experiments, we confirmed that the parameters used by *de novo* could assemble the reads into separate contigs, resulting in the expected viruses.

#### Unspiked Experiments

To validate this method for field studies, we tested unknown mixtures of viral isolates present in the supernatant of RD cells infected with extracts from stools and sewage samples. Ten samples from stools and ten samples from sewage were used, following the protocols described in the materials and methods section. Full genomic sequences were identified for 8 viruses present in RD supernatants from stool samples and 5 viruses present in those from sewage samples. However, for certain viruses, contigs covering only partial genomic sequences could be obtained. In these cases, we could use the VP1 contigs to identify the viruses present. In cell supernatants of infected cells, we were able to recover 1 to 2 viruses per stool sample and 2 to 7 viruses per sewage sample (**Table 5**). Among twenty samples, we were able to find 48 enteroviruses based on the VP1 region. For 21 viruses, more than 6100 nt per genome could be determined, whereas, for 27 other viruses, genomic contigs were not as long. The genome of 18 viruses was higher than 90% in length. Overall, 41 EV-Bs, 3 EV-As, 3 EV-Cs, and no EV-Ds were identified.

## Discussion

The goal of this research was to genetically sequence the entire genome for all enteroviruses (EV-A, -B, -C, and –D) present in a given sample, using a simple method for detection and genetic characterization. To accomplish this, we developed an RT-PCR assay where we designed degenerate primers targetting conserved regions of the genome, which allowed the DNA fragments to be amplified, followed by NGS. This paper described our approach and demonstrated the feasibility of our methods to successfully identify EV in mixtures.

The effectiveness of this assay was achieved by selecting two primer sets per enterovirus species that generated two overlapping fragments decreasing labor, time, and cost per sample tested. The first generic primer set (C004-F and CRE-R) corresponds to two conserved regions for all enteroviruses (-A through -D) and was capable of successfully amplifying the first half of the genome (∼4500 nt). The second half of the genome was amplified using generic primers that were designed to specifically target all viruses within the respective species (EV-A, -B, -C, and -D). We were able to amplify nearly the entire genome for all enteroviruses, using only five PCR mixtures. The sensitivity of the primers was determined and the EV-A primers appeared to be more sensitive than the others (**Table 3**). However, the primers selected for this project were sensitive enough to detect all enteroviruses and specific enough to detect only one species type in the presence of the other species.

To validate our sequencing method, we performed it using twenty RD isolate samples from human stools and sewage concentrates. The consensus lengths after *de novo* assembly were obtained for the ten isolate samples from stool tested, indicating that the method was sensitive enough to determine the entire genome. The results were different for sewage because mixtures of strains appeared to be more complex (up to 7 different strains) than those present in stools (two strains maximum). Only three of the ten isolate samples resulted in sequencing data for the whole genome. However, the sequencing data for the other seven provided long genomic contigs and the coverage necessary to identify the type of virus(es) present using VP1 sequences (Nix et al., 2006). Additionally, we effectively recovered the four viruses used to produce the known mixtures described in **Table 4** and the enterovirus field mixtures from **Table 5**. Being able to differentiate viruses in mixtures is important and in this study, and in a previous study (Bessaud et al., 2016), we have found that *de novo* assembly is able to properly construct contigs when mixed viruses are genetically divergent (i.e., when they do not share identical genetic sequences). However, recombination can create genomes that, together, are clearly divergent in some genomic regions and completely identical in others. In this case, *de novo* assembly can fail to build full-length contigs because it is impossible for the software to determine which reads are from virus 1 or from virus 2. This limitation is not related to our method used to generate DNA from RNA genomes, but it is due to the fact that most NGS methods (i.e., Illumina) cannot deal with long DNA fragments, requiring the shearing of the DNA amplicons prior to sequencing. Other NGS methods (such as PacBio or MinION) can sequence long DNA fragments and could be used to overcome the assembly problems but these methods have a high error rate compared to Illumina. Therefore, there is a balance between the accuracy of the sequences and the length of the contigs that can be generated in cases of mixed recombinant genomes. Nonetheless, this challenge did not compromise the main objective of our method, which was to allow the identification of the enterovirus lineages found in a given sample. The typing of EVs relies on their capsid sequence and intertypic recombination is very uncommon inside this genomic region, the contigs generated through *de novo* assembly generally span all the capsid-encoding region.

Research has shown the importance of analyzing the complete genome for enteroviruses (Oberste et al., 2004; Joffret et al., 2012) because nucleotidic differences and inter and intra-typic recombination events in non-structural regions differentiate types and lineages (Lukashev et al., 2003; Simmonds, 2006; Combelas et al., 2011). The amplification and sequencing of whole genomic sequences of strains belonging to EV-A, -B, and -C species using generic and specific primers were successfully performed (Tan et al., 2015; Bessaud et al., 2016; Montmayeur et al., 2017; Sahoo et al., 2017). One particular study aimed to isolate polioviruses using random amplification and NGS to better optimize current protocols for whole genome sequencing and identification of a variety of vaccine-derived polioviruses (Montmayeur et al., 2017). Although these methods were able to recover the entire genome for a given type or species, they were not able to sequence mixtures of several human enteroviruses in a single run like our described research. However, a recent study focused on detecting polioviruses and non-polio enteroviruses in cellular supernatants infected with sewage concentrates using NGS and random primers or specific primers targeting polioviruses (Majumdar et al., 2018).

Contrary to these previous studies, our assay was designed to specifically amplify enterovirus of species A to D, capturing the diversity of EVs. Because the assay includes species-specific primers, we can increase the amplification of the respective targeted species. This is not the case with random primers that cannot be ensured to capture all enteroviruses within a given species. In addition, the assay can be simplified to target one particular species. Although strategies based on random amplification have the advantage of being able to amplify sequences of viruses that are unexpected or unknown, they have the disadvantage of decreasing the number of relevant reads since the sequence of non-viral origins is also amplified and sequenced. Our strategy is more suitable to specifically amplify the relevant sequences, limiting the requested depth of coverage, which favors multiplexing and thus reducing the cost per genome and per sample.

In conclusion, the method described in this study enables the specific identification of all enteroviruses present in samples during a single sequencing run. This type of assay would be useful when analyzing human and environmental field samples as indicated in our results.

This contributes to the study of EV diversity and ecosystem within given populations. In addition, this method provided a way to collect full genome sequencing data from mixtures of enteroviruses within the four species A to D that were present in cellular supernatants. These data become a necessity for surveillance purposes and when studying the relationships between the genetic characteristics, including the mosaic features of their genomes acquired through frequent intra- and intertypic recombination and their biological properties. Indeed, mutations and recombination events can be implicated in reintroducing virulence factors and have been involved in the evolution of enteroviruses (Riquet et al., 2008; Jegouic et al., 2009; Joffret et al., 2012; Holmblat et al., 2014).

## Author Contributions

M-LJ, PP, and FD conceived and designed the experiments and analyzed the data. M-LJ and PP performed the experiments. RR and J-MH contributed to samples and experiments in Madagascar. PP and M-LJ wrote the manuscript. MB, RR, J-MH, and FD critically revised.

## Conflict of Interest Statement

The authors declare that the research was conducted in the absence of any commercial or financial relationships that could be construed as a potential conflict of interest.

## Footnotes

1 https://www.european-virus-archive.com

## References

[B1] Bessaud M. Sadeuh-Mba S. A. Joffret M.-L. Razafindratsimandresy R. Polston P. Volle R. (2016). Whole genome sequencing of *Enterovirus* species C isolates by high-throughput sequencing: development of generic primers. *Front. Microbiol.* 7:1294. 10.3389/fmicb.2016.01294 27617004 PMC4999429

[B2] Blomqvist S. Roivainen M. (2016). Isolation and characterization of enteroviruses from clinical samples. *Methods Mol. Biol.* 1387 19–28. 10.1007/978-1-4939-3292-4_3 26983729

[B3] Caro V. Guillot S. Delpeyroux F. Crainic R. (2001). Molecular strategy for ‘serotyping’of human enteroviruses. *J. Gen. Virol.* 82 79–91. 10.1099/0022-1317-82-1-79 11125161

[B4] Combelas N. Holmblat B. Joffret M. L. Colbere-Garapin F. Delpeyroux F. (2011). Recombination between poliovirus and coxsackie A viruses of species C: a model of viral genetic plasticity and emergence. *Viruses* 3 1460–1484. 10.3390/v3081460 21994791 PMC3185806

[B5] Holmblat B. Jégouic S. Muslin C. Blondel B. Joffret M.-L. Delpeyroux F. (2014). Nonhomologous recombination between defective poliovirus and coxsackievirus genomes suggests a new model of genetic plasticity for picornaviruses. *mBio* 5:e1119-14. 10.1128/mBio.01119-14 25096874 PMC4128350

[B6] Jegouic S. Joffret M. L. Blanchard C. Riquet F. B. Perret C. Pelletier I. (2009). Recombination between polioviruses and co-circulating Coxsackie A viruses: role in the emergence of pathogenic vaccine-derived polioviruses. *PLoS Pathog.* 5:e1000412. 10.1371/journal.ppat.1000412 19412342 PMC2669712

[B7] Joffret M. L. Jegouic S. Bessaud M. Balanant J. Tran C. Caro V. (2012). Common and diverse features of cocirculating type 2 and 3 recombinant vaccine-derived polioviruses isolated from patients with poliomyelitis and healthy children. *J. Infect. Dis.* 205 1363–1373. 10.1093/infdis/jis204 22457288

[B8] Lukashev A. N. Lashkevich V. A. Ivanova O. E. Koroleva G. A. Hinkkanen A. E. Ilonen J. (2003). Recombination in circulating enteroviruses. *J. Virol.* 77 10423–10431. 10.1128/jvi.77.19.10423-10431.2003 12970427 PMC228507

[B9] Majumdar M. Klapsa D. Wilton T. Akello J. Anscombe C. Allen D. (2018). Isolation of vaccine-like poliovirus strains in sewage samples from the United Kingdom. *J. Infect. Dis.* 217 1222–1230. 10.1093/infdis/jix667 29309594

[B10] Montmayeur A. M. Ng T. F. Schmidt A. Zhao K. Magana L. Iber J. (2017). High-throughput next-generation sequencing of polioviruses. *J. Clin. Microbiol.* 55 606–615. 10.1128/JCM.02121-16 27927929 PMC5277531

[B11] Nijhuis M. van Maarseveen N. Schuurman R. Verkuijlen S. de Vos M. Hendriksen K. (2002). Rapid and sensitive routine detection of all members of the genus enterovirus in different clinical specimens by real-time PCR. *J. Clin. Microbiol.* 40 3666–3670. 10.1128/JCM.40.10.3666-3670.2002 12354863 PMC130891

[B12] Nix W. A. Oberste M. S. Pallansch M. A. (2006). Sensitive, seminested PCR amplification of VP1 sequences for direct identification of all enterovirus serotypes from original clinical specimens. *J. Clin. Microbiol.* 44 2698–2704. 10.1128/JCM.00542-06 16891480 PMC1594621

[B13] Oberste M. S. Maher K. Kilpatrick D. R. Flemister M. R. Brown B. A. Pallansch M. A. (1999a). Typing of human enteroviruses by partial sequencing of VP1. *J. Clin. Microbiol.* 37 1288–1293. 10203472 10.1128/jcm.37.5.1288-1293.1999 PMC84754

[B14] Oberste M. S. Maher K. Kilpatrick D. R. Pallansch M. A. (1999b). Molecular evolution of the human enteroviruses: correlation of serotype with VP1 sequence and application to picornavirus classification. *J. Virol.* 73 1941–1948. 9971773 10.1128/jvi.73.3.1941-1948.1999 PMC104435

[B15] Oberste M. S. Maher K. Pallansch M. A. (2004). Evidence for frequent recombination within species human enterovirus B based on complete genomic sequences of all thirty-seven serotypes. *J. Virol.* 78 855–867. 10.1128/JVI.78.2.855-867.2004 14694117 PMC368751

[B16] Poh C. L. Tan E. L. (2010). “Detection of enteroviruses from clinical specimens,” in *Diagnostic Virology Protocols*, eds Stephenson J. R. Warnes A. (Berlin: Springer), 65–77. 10.1007/978-1-60761-817-1_5

[B17] Riquet F. B. Blanchard C. Jegouic S. Balanant J. Guillot S. Vibet M. A. (2008). Impact of exogenous sequences on the characteristics of an epidemic type 2 recombinant vaccine-derived poliovirus. *J. Virol.* 82 8927–8932. 10.1128/JVI.00239-08 18579607 PMC2519664

[B18] Sadeuh-Mba S. A. Bessaud M. Massenet D. Joffret M. L. Endegue M. C. Njouom R. (2013). High frequency and diversity of species C enteroviruses in Cameroon and neighboring countries. *J. Clin. Microbiol.* 51 759–770. 10.1128/JCM.02119-12 23254123 PMC3592076

[B19] Sahoo M. K. Holubar M. Huang C. Mohamed-Hadley A. Liu Y. Waggoner J. J. (2017). Detection of emerging vaccine-related polioviruses by deep sequencing. *J. Clin. Microbiol.* 55 2162–2171. 10.1128/JCM.00144-17 28468861 PMC5483918

[B20] Silva P. A. Diedrich S. de Paula Cardoso D. D. Schreier E. (2008). Identification of enterovirus serotypes by pyrosequencing using multiple sequencing primers. *J. Virol. Methods* 148 260–264. 10.1016/j.jviromet.2007.10.008 18082902

[B21] Simmonds P. (2006). Recombination and selection in the evolution of picornaviruses and other mammalian positive-stranded RNA viruses. *J. Virol.* 80 11124–11140. 10.1128/JVI.01076-06 16956935 PMC1642140

[B22] Tan L. V. Tuyen N. T. K. Thanh T. T. Ngan T. T. Van H. M. T. Sabanathan S. (2015). A generic assay for whole-genome amplification and deep sequencing of enterovirus A71. *J. Virol. Methods* 215 30–36. 10.1016/j.jviromet.2015.02.011 25704598 PMC4374682

